# Global spread and antimicrobial resistance of *Aeromonas hydrophila* in aquatic food animals: a systematic review and meta-analysis

**DOI:** 10.1038/s41598-025-14498-8

**Published:** 2025-08-04

**Authors:** Saharuetai Jeamsripong, Justice Opare Odoi, Manoj Kumar Shahi, Saran Anuntawirun, Nawaphorn Roongrojmongkhon, Anyarat Thiptara

**Affiliations:** 1 Research Unit in Microbial Food Safety and Antimicrobial Resistance, Department of Veterinary Public Health, Faculty of Veterinary Science, Chulalongkorn University, Bangkok, Thailand; 2 Department of Veterinary Public Health, Faculty of Veterinary Science, Chulalongkorn University, Bangkok, Thailand; 3 Division of Animal Health, Animal Research Institute, Council of Scientific and Industrial Research, Accra, Ghana; 4 Veterinary Research and Development Center (Upper Southern Region), Thung-Song, Nakhon-Si-Thammarat, Thailand

**Keywords:** *Aeromonas hydrophilla*, Antimicrobial resistance, ESBL, Multidrug resistance, Virulence factors, Natural hazards, Infectious diseases

## Abstract

*Aeromonas hydrophila* is a common zoonotic agent in aquatic environments that causes gastroenteritis and wound infections in both humans and animals through foodborne and hospital-acquired infection. Antimicrobial resistance (AMR) combination with virulence factors enhances treatment challenging. The prevalence and AMR of *Aeromonas hydrophila* have been increasingly reported, posing a significant threat to both animal and public health. This systematic review and meta-analysis aimed to determine the prevalence of *A. hydrophila* and its resistance to aquatic food animals. A comprehensive search for relevant studies was conducted on Google Scholar, PubMed, ScienceDirect, and Scopus, following the PRISMA guidelines, covering studies from January 2020 to December 2024. The quality of the included publications was evaluated using the Joanna Briggs Institute critical appraisal tool. Differences in the prevalence and AMR of *A. hydrophila* were assessed using a random-effect model. A total of 14,077 studies were screened, and 14 publications were included. Bacterial isolation of *A. hydrophila* was achieved using various standard protocols, involving culture on Rimler-Shotts (RS) agar and Tryptic Soy Agar (TSA), with or without antimicrobials. Subsequent biochemical identification confirmed the isolates. The pool prevalence of *A. hydrophila* (30.7%, 95% C.I.:17.0-46.3%), and the distribution of virulence genes were 71.2%. The most common resistance observed to penicillin (80.7%), oxytetracycline (69.9%) and macrolides (67.8%). The most prevalent AMR genes identified were *bla*_TEM_ (67.0%), followed by *tetA* (63.7%). The increasing presence of *A. hydrophila* in aquaculture suggests a considerable risk of disease. The prevalence of both *A. hydrophila* and AMR was higher in Africa than in Asia, indicating regional variations in the AMR pattern. However, monitoring and surveillance of *A. hydrophila* remained limited. A major limitation of this study was the heterogeneity in effect estimates across the selected studies. Nonetheless, the quality assessment conducted indicated that this variability did not compromise the consistency or reliability of the findings.

**Supplementary Information:**

The online version contains supplementary material available at 10.1038/s41598-025-14498-8.

## Introduction

*Aeromonas hydrophila* presents a significant concern due to its zoonotic nature, causing gastroenteritis and wound infections in humans, with aquatic animals serving as a key source of exposure. Concurrently, its widespread prevalence and demonstrated phenotypic and genotypic resistance impact on the aquaculture industry globally, leading to substantial disease burdens and economic losses.

 Aquatic food animals, such as fish, shrimp, and shellfish, are becoming increasingly crucial for global food security, providing high-quality protein and essential bioavailable micronutrients^1^. In Southeast Asian countries, aquaculture is a key economic sector, particularly in aquatic food farming. According to a FAO report, global fisheries and aquaculture production reached a record 214 million tons in 2020, valued at approximately USD 424 billion, making a more than 60% increase compared to average production levels in the 1990s^2^. This growth underscores aquaculture expanding’s role in meeting the increasing global protein demand and ensuring food security. However, the intensification of aquaculture, driven by increasing global seafood consumption, also presents significant public health challenges. The widespread use of antimicrobials in aquaculture has led to the emergence of antimicrobial resistance (AMR)^3^. As reliance on fisheries and aquaculture continues to grow, addressing the challenges posed by AMR and pathogenic bacteria in seafood becomes increasingly critical.

The widespread use of antimicrobials in aquaculture to prevent and treat bacterial infections, combined with contamination from terrestrial sources, such as livestock production systems and sewage, has turned aquaculture environment hotspots for AMR^4^. One significant pathogen in aquaculture is *A. hydrophila*, a Gram-negative bacterium commonly found in aquatic environments^5^. This bacterium inflicts significant financial damage in aquaculture through diseases like Motile Aeromonas Septicemia (MAS) on fish, causing massive fatalities. More importantly, *A. hydrophila* is emerging as a key contributor to the spread of AMR genes, threatening the effectiveness of antimicrobials in both human and veterinary medicine. With its capacity to cause disease and its rising resistance to widely use antimicrobials, *A. hydrophila* presents considerable dangers to both human and animal health^6^. Studies have shown that *A. hydrophila* can harbor multiple resistance genes, including those for β-lactamases, tetracycline, and quinolones, which are commonly used antimicrobials both in human medicine and aquaculture^7,8^. The presence of multidrug-resistant (MDR) *A. hydrophila* strains in seafood has severe food safety concerns, as the bacterium can be transmitted to humans through the consumption of contaminated seafood or direct contact with water sources where the pathogen thrives. This issue is further compounded by the fact that shrimp, fish, and shellfish, may serve as a key vector for the transmission of resistant strains to consumers. As a result, *A. hydrophila* has emerged as a significant public health concern, especially due to its potential to transfer resistance genes across bacterial populations, highlighting the urgent need to control its spread in seafood and the broader environment.

Beyond AMR, various virulence factors that enhance the ability of *A. hydrophila* to cause disease in its host have been identified. The combination of virulence factors with AMR greatly enhances the pathogenic potential of *A. hydrophila*. This bacterium is known for causing a range of infections in humans, including gastroenteritis and wound infections. In fact, *A. hydrophila* has been implicated in foodborne disease outbreaks as well as hospital-acquired infections and gastroenteritis^6,9^. Given their increasing presence in aquatic systems, continuous surveillance and up-to-date information on the prevalence, association with disease burden, AMR, and virulence factors in seafood-associated *A. hydrophila* isolates are essential for accurately assessing the risks they pose to public health.

Individual studies frequently lack a comprehensive view, but this research bridges that gap by aggregating data from diverse geographical regions, providing a genuine global understanding of the pathogen’s spread and AMR. Therefore, this systematic review and meta-analysis were to assess the global prevalence of *A. hydrophila* in seafood animal species, while also exploring its AMR patterns and associated virulence factors. The review addressed the following research questions: (1) What was the prevalence of *A. hydrophila* in aquatic food animals, including fish, shrimp, and shellfish? (2) What were the phenotypic and genotypic characteristics of AMR in *A. hydrophila* strains? and (3) What virulence factors were commonly associated with *A. hydrophila* strains affect public health and the sustainability of aquaculture. By comparing trends in AMR and virulence factors across various aquatic food animals and geographical regions, this study critically investigates these aspects, aiming to provide essential insights for informing strategies that enhance public health outcomes and promote the sustainability of aquaculture practices.

## Materials and methods

### Protocol registration

This systematic review was developed and conducted following the Preferred Reporting Items for Systematic Reviews and Meta-Analysis (PRISMA) guidelines^10^. The systematic review was registered and certain aspects of this systematic review and meta-analysis were conducted in accordance with the previously established procedure^11^.

### Data searches

Comprehensive systematic review searches were conducted in Google Scholar (https://scholar.google.com/), PubMed (https://pubmed.ncbi.nlm.nih.gov/), ScienceDirect (https://www.sciencedirect.com/), and Scopus (https://www.scopus.com/) of study published from January 2020 to December 2024. This study primarily used Scopus for the main literature search due to its extensive indexing, while ScienceDirect served as a supplementary tool, mainly for directly accessing full texts from identified Elsevier journals or for initial, targeted keyword searches.

The search term was following search terms: ((“*Aeromonas hydrophilla*” OR “*A. hydrophila*”) AND (“antibiotic” OR “antibiogram” OR “antimicrobial resistance” OR “resistance” OR “multidrug resistance” OR “antimicrobial susceptibility testing” OR “multidrug resistance OR “genotypic determinants” OR “genotype” AND (“virulence factors” OR “virulence genes”) AND (“fish” OR “shellfish” OR “shrimp” OR “seafood”), with no language restrictions (Supplementary Table S1.). To ensure a comprehensive collection of studies, several keyword combinations were used for each relevant topic area. This strategy was designed to include as many studies as possible, covering a broad spectrum of research on *A. hydrophila*, AMR, and different aquatic types. The search was performed in December 2024.

To guide the protocol writing and registration process, the seventeen-item Preferred Reporting Items for Systematic Reviews and Meta-Analyses Protocols 2015 checklist was followed^10,12^. Finally, the results of the review were presented using the Preferred Reporting Items for Systematic Reviews and Meta-Analyses (PRISMA) 2020 checklist.

### Definitions

AMR refers to the ability of *A. hydrophila* to survive and grow in the presence of antimicrobial agents that would normally inhibit or kill them. MDR is defined as the resistance of *A. hydrophila* to three or more classes of antimicrobial agents^13^. Seafood encompasses a wide range of edible aquatic animals, including fish, shrimp, and shellfish, whether harvested from the wild or farmed through aquaculture. In the context of this review, seafood is the source from which *A. hydrophila* strains are isolated for the study of AMR and virulence factors.

### Eligibility criteria

For this systematic review, eligibility was limited to peer-reviewed original research articles published in reputable scientific journals, each assigned to a DOI.These journals had to be indexed in recognized databases such as Google Scholar, PubMed, Science Direct, and Scopus. Selected articles focused on evaluating the prevalence, virulence potential and AMR of *A. hydrophila* isolated from seafood species, regardless of whether the seafood was wild-caught or farmed in aquaculture. Only studies that were accessible for comprehensive analysis and adhered to stringent reporting standards were deemed eligible. It was essential that these studies provided clear descriptions or appropriate references for the methodologies employed in bacterial isolation and identification, antimicrobial susceptibility testing (AST), and virulence factor detection. Additionally, the findings, discussions, and conclusions in these studies had to be clearly presented and structured in a manner that allowed for thorough evaluation and critical appraisal. These stringent criteria were consistently applied during the selection process to ensure that only the most relevant and high-quality studies were included in the review.

### Inclusion criteria

The inclusion criteria were determined based on the methodological guidance for systematic reviews of observational epidemiological studies reporting prevalence data following CoCoPop mnemonic (condition, context, and population)^14^. The inclusion criteria for this review encompassed primary, cross-sectional studies published in English in peer-reviewed journals between January 2020 and December 2024. These studies had to involve *A. hydrophila* isolates from various aquatic animal species commonly consumed or associated with seafood-related illnesses, specifically those intended for human consumption. Furthermore, studies were included required to measure outcomes related to the detection and confirmation of *A. hydrophila* and provide information on both its phenotypic and genotypic AMR.

### Exclusion criteria

Exclusion criteria were applied to studies if they presented insufficient data for extraction (e.g., lacking full-text access or being non-free full-text), or if they were review articles, experimental studies, conference proceedings, case reports, case series, or letters. Additionally, studies were excluded if focused on *A. hydrophila* isolates from non-aquatic animals or aquatic animals not primarily intended for human consumption, or if they explored only environmental samples from aquaculture without a direct link to aquatic animal isolates. Finally, studies were excluded if they lacked core outcome data, specifically the observable prevalence of *A. hydrophila* or comprehensive information on both phenotypic and genotypic AMR of *A. hydrophila*, and any duplicate studies identified across databases were systematically removed.

### Data reliability

To ensure data reliability, a rigorous methodology was applied, requiring a minimum sample size of at least 20 isolates to minimize between-study heterogeneity, as previously suggested^15^. Studies containing fewer than 20 isolates may disproportionately influence the meta-analysis due to inherent variability and potential bias.

### Study selection procedures

Relevant studies were retrieved from electronic database searches were imported and stored in EndNote 9 (Clarivate Analytics, PA, USA). The study selection process included the removal of duplicates, screening titles and abstracts, and examining full texts based on eligibility criteria. Two authors (MKS and SA) independently screened the titles and abstracts of the articles against the inclusion criteria. Any disagreement between reviewers was verified by consultation with a third reviewer (SJ). To assess the level of agreement between the authors, inter-rater reliability was calculated using the Cochrane Handbook for Systematic Reviews (Higgins et al., 2019). A kappa value of 0.75 or higher was considered to indicate excellent agreement. Only studies that fully met the initial screening were selected and reviewed in full by two independent authors (SJ and SA). If further clarification was needed regarding eligibility, the other authors were consulted. Disagreements over study inclusion were resolved through discussion.

### Risk of bias and quality assessment

The risk of bias in eligible studies was assessed using the Joanna Briggs Institute (JBI) critical appraisal tools, designed for cross-sectional study designs^14^ (Supplementary Table S2). For cross-sectional studies, these tools included nine categories on the checklist for studies reporting prevalence, which is focused on inclusion criteria, reliability and accuracy of measurement, including study subjects, adequacy of response, methods used for identification, standard, and statistical analysis. The risk of bias was classified based on a total score of 9. Studies were categorized as follows: low risk of bias: 7–9 scores, moderate risk of bias: 4–6 scores, and high risk of bias: 3 scores. Only studies with a total score of 7 or higher were included in the quantitative analysis to ensure the reliability and validity of the results. Two authors (SA and JOO) independently assessed the risk of bias. Any disagreements were resolved through discussion with a third investigator (SJ).

### Data analysis

To determine the prevalence of *A. hydrophila* and the phenotypic and genotypic resistance of *A. hydrophila*, the number of positive samples or isolates was calculated as a proportion of the total samples or isolates tested. The prevalence of *A. hydrophila* and resistant *A. hydrophila* were estimated using meta-analysis with a random-effects model. Results were presented as a pooled prevalence estimate with a 95% confidence interval (C.I.). Heterogeneity was assessed virtually using forest plots with pooled estimates.

Heterogeneity among eligible studies was assessed based on Cochran’s Q test (χ²) with a *p*-value < 0.10. The variability between studies was classified using Higgins and Thompson’s *I²* index, which classified studies as having 0% (no heterogeneity), 25–50% (low heterogeneity), 50–75% (moderate heterogeneity), and > 75% (high heterogeneity)^16^. Subgroup meta-analyses and sensitivity analyses were performed to explore potential sources of statistical heterogeneity based on sample characteristics, including publication year, geographical distribution, aquatic animal types, and antimicrobial classes. Pooled prevalence estimates were derived from relevant data in at least two individual studies enhancing the robustness of the findings.

Publication bias was assessed visually using funnel plots and tested with Egger’s test with a *p*-value of < 0.05 indicated potential bias. A contour-enhanced funnel plot was used to determine whether funnel plot asymmetry was due to publication bias or other factors. If asymmetry was found to be due to publication bias. All statistical analyses were conducted using STATA (Version 18, StataCorp LLC, College Station, TX, USA).

## Results

### Search results

**Fig. 1:**
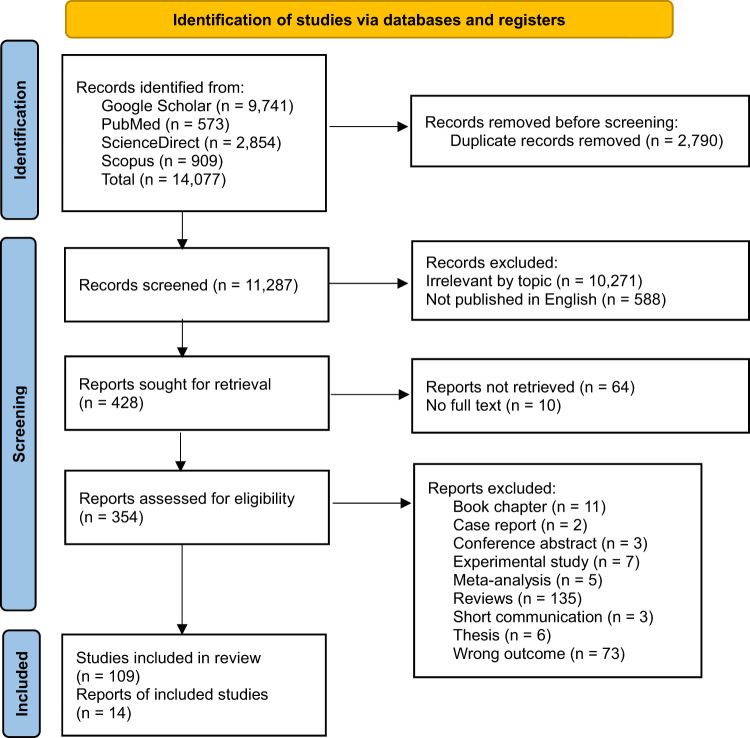
A PRISMA flowchart outlining the study selection process and literature search. Multiple databases were systematically reviewed using predefined search strategies to identify eligible studies focusing on prevalence and resistance of *A. hydrophila*.

A total of 14,077 studies were identified through Google Scholar (*n* = 9,741), PubMed (*n* = 573), ScienceDirect (*n* = 2,854), and Scopus (*n* = 909). After removing duplicates, a total of 11,287 studies were considered. Subsequently, studies were excluded for the following reasons: irrelevant topic (*n* = 10,271), not published in English (*n* = 588), and those that could not be retrieved or lacked full text (*n* = 74). The remaining 354 articles were screened, and finally 14 articles were selected for quantitative analysis (Fig. 1).

### Study characteristics

A total of 14 published articles involving 2,094 samples were included (Table 1). The sample sizes of the primary studies varied, ranging from 71 to 500 samples. The characteristics of eligible studies are summarized. Most of the studies were indexed in Google Scholar (*n* = 8), followed by PubMed (*n* = 4) and Scopus (*n* = 2). Most of the studies originated from Asia (*n* = 8) and Africa (*n* = 6). All studies used cross-sectional research designs. The samples in the primary studies were collected from various fish species (e.g., catfish, carp, tilapia, tuna, milkfish, mugil cephalus, and mugil fish) and shrimp (e.g., riceland prawn). Probability sampling techniques were employed to select a representative sample. Most specimens were internal organs (*n* = 9), followed by gills (*n* = 5), and carcass rinse samples (*n* = 4), although studies included different sample types. Additionally, two studies contained water samples for the analysis.

**Table 1:** The characteristics of the studies included in the systematic review and meta-analysis.

ID	Author	Year	Duration of study	Country	Continent	Type of sample	Sample specimen*
1	Thongkao and Sudjaroen^17^	2019	Jan - Apr 2019	Thailand	Asia	*Macrobrachium lanchesteri* and Kung Ten	Whole shrimp
2	Azzam-Sayuti et al.^18^	2021	Feb - Sep 2019	Malaysia	Asia	Walking catfish, tilapia, striped catfish	Kidney, liver, and spleen
3	Bardhan and Abraham^19^	2021	Apr 2018 - Mar 2019	India	Asia	Carp	Meat with skin
4	Abd El-Tawab et al.^20^	2021	Jan - Oct 2019	Egypt	Africa	Nile tilapia and mugil fish	liver, kidneys, spleen, heart, anterior intestine, and gills
5	Saleh et al.^21^	2021	Apr - Sep 2019	Egypt	Africa	Nile tilapia	Liver, spleen, intestine, and gills
6	Eid et al. (a)^22^	2022	Jul 2019 - Sep 2020	Egypt	Africa	Mugil cephalus	Surface, gills, liver, kidney, spleen and water
7	Eid et al. (b)^23^	2022	2022	Egypt	Africa	Nile tilapia	Gill and internal organ
8	Fikri et al.^24^	2022	Mar - Jul 2020	Indonesia	Asia	Milkfish	Gill
9	Goudarztalejerdi et al.^25^	2022	2022	Iran	Asia	Carp (*Cyprinidae*)	Fish surface and kidney
10	Morshdy et al.^26^	2022	NA	Egypt	Africa	Tilapia, mugel, tuna, saurus, pagrus and shrimp	Meat
11	U-taynapun and Chirapongsatonkul^27^	2022	2016–2020	Thailand	Asia	Nile tilapia	Brain and liver
12	Morshdy et al.^28^	2023	NA	Egypt	Africa	Mullet, catfish, lizard and coralfish	Meat
13	Thaotumpitak et al.^29^	2023	Oct - Nov 2020	Thailand	Asia	Nile tilapia	Carcass rinse, intestine, mucus, liver and kidney
14	Thi and Dung et al.^30^	2023	Jan 2020 - Apr 2021	Vietnam	Asia	Catfish (*Pangasius*)	Kidney, liver, and spleen

Note: Some eligible studies may have included more than one type of sample.

The assessment of the prevalence of *A. hydrophila* was conducted across different years in 2020 (*n* = 1), 2021 (*n* = 4), 2022 (*n* = 6), and 2023 (*n* = 3), respectively (Table 2). The data only were retrieved from Africa (Egypt-6) and Asia (Thaialnd-3, Indonesia-1, Malysia-1, Iran-1, India-1, and Vietnam-1). The number of samples varied from 71 to 828, with positive isolates ranging from 17 to 187. Different protocols have been used for the bacterial isolation of *A. hydrophila*, including methods from the U.S. Food and Drug Administration Bacteriological Analytical Manual (U.S. FDA BAM), Public Health England (PHE), and Bergey’s Manual of Systematic Bacteriology. Aeromonas selective media, Rimler-Shotts (RS) and TSA, were commonly used, with or without antimicrobials such as ampicillin and novobiocin. For the confirmation of *A. hydrophila*, biochemical identification tests were employed, including Aerokey II, API 20E, and various assays for arginine decarboxylase, β-galactosidase, bile esculin, catalase, citrate, cyanide, gelatinase, Gram staining, growth in 0% and 6.5% NaCl, hydrolysis, hydrogen sulfide, indole, lipase, lysine decarboxylase, mannitol, methyl red-Voges-Proskauer (MR-VP), motility, ornithine decarboxylase, oxidase, pigment formation, potassium, protease, sugar fermentation, sulfide, triple sugar iron (TSI), and urease tests.

**Table 2:** Summary of overall prevalence of *A. hydrophila*.

ID	Author	Year	Duration of study	Country	Continent	No. of sample	Prev (%)	95% C.I.
1	Abd El-Tawab et al.^20^	2021	Jan - Oct 2019	Egypt	Africa	100	65.0	55.3–74.1
2	Saleh et al.^21^	2021	Apr - Sep 2019	Egypt	Africa	500	37.4	33.2–41.7
3	Eid et al. (a)^22^	2022	Jul 2019 - Sep 2020	Egypt	Africa	100	24.0	16.1–32.9
4	Eid et al. (b)^23^	2022	2022	Egypt	Africa	100	65.0	55.3–74.1
5	Morshdy et al.^26^	2022	NA	Egypt	Africa	150	12.0	7.2–28.5
6	Morshdy et al.^28^	2023	NA	Egypt	Africa	100	20.0	12.7–28.5
	Prevalence in Africa						36.0	18.6–55.5
7	Thongkao and Sudjaroen^17^	2019	Jan - Apr 2019	Thailand	Asia	120	16.7	10.5–23.9
8	Azzam-Sayuti et al.^18^	2021	Feb - Sep 2019	Malaysia	Asia	270	9.3	6.1–13.0
9	Bardhan and Abraham^19^	2021	Apr 2018 - Mar 2019	India	Asia	71	43.7	32.3–55.4
10	Goudarztalejerdi et al.^25^	2022	2022	Iran	Asia	100	42.0	32.5–51.8
11	U-taynapun and Chirapongsatonkul^27^	2022	2016–2020	Thailand	Asia	250	6.8	4.0-10.3
12	Fikri et al.^24^	2022	Mar - Jul 2020	Indonesia	Asia	153	22.9	16.5–29.9
13	Thaotumpitak et al.^29^	2023	Oct - Nov 2020	Thailand	Asia	828	3.1	2.1–4.4
14	Thi and Dung et al.^30^	2023	Jan 2020 - Apr 2021	Vietnam	Asia	80	92.5	85.5–97.4
	Prevalence in Africa						26.9	8.5–50.7
	Grand total prevalence						30.7	17.0-46.3

Additionally, molecular techniques using PCR with specific genes (e.g., *act*; *aeroH*; *aer*; *aerA*) and 16 S rRNA were conducted for identification of *A. hydrophila*. For AST, most studies used the disk diffusion method (*n* = 13) and agar dilution (*n* = 1), along with standard methods such as those from the National Committee for Clinical Laboratory Standards (NCCLS), European Committee on Antimicrobial Susceptibility Testing (EUCAST), and Clinical and Laboratory Standards Institute (CLSI 2012, 2014–2016, M45, and M100). The control strains used in AST were identified as *A. hydrophila* (ATCC 7966) (*n* = 3), *Escherichia coli* (ATCC 25922) (*n* = 1), and a combination of *Staphylococcus aureus* (ATCC 29213), *E. coli* (ATCC 25922), and *Pseudomonas aeruginosa* (ATCC 27853) (*n* = 1). In contrast, most publications (*n* = 9) did not mention the use of a control strain.

### Pooled prevalence of *A. hydrophila* stratified by geographical distribution

**Fig. 2:**
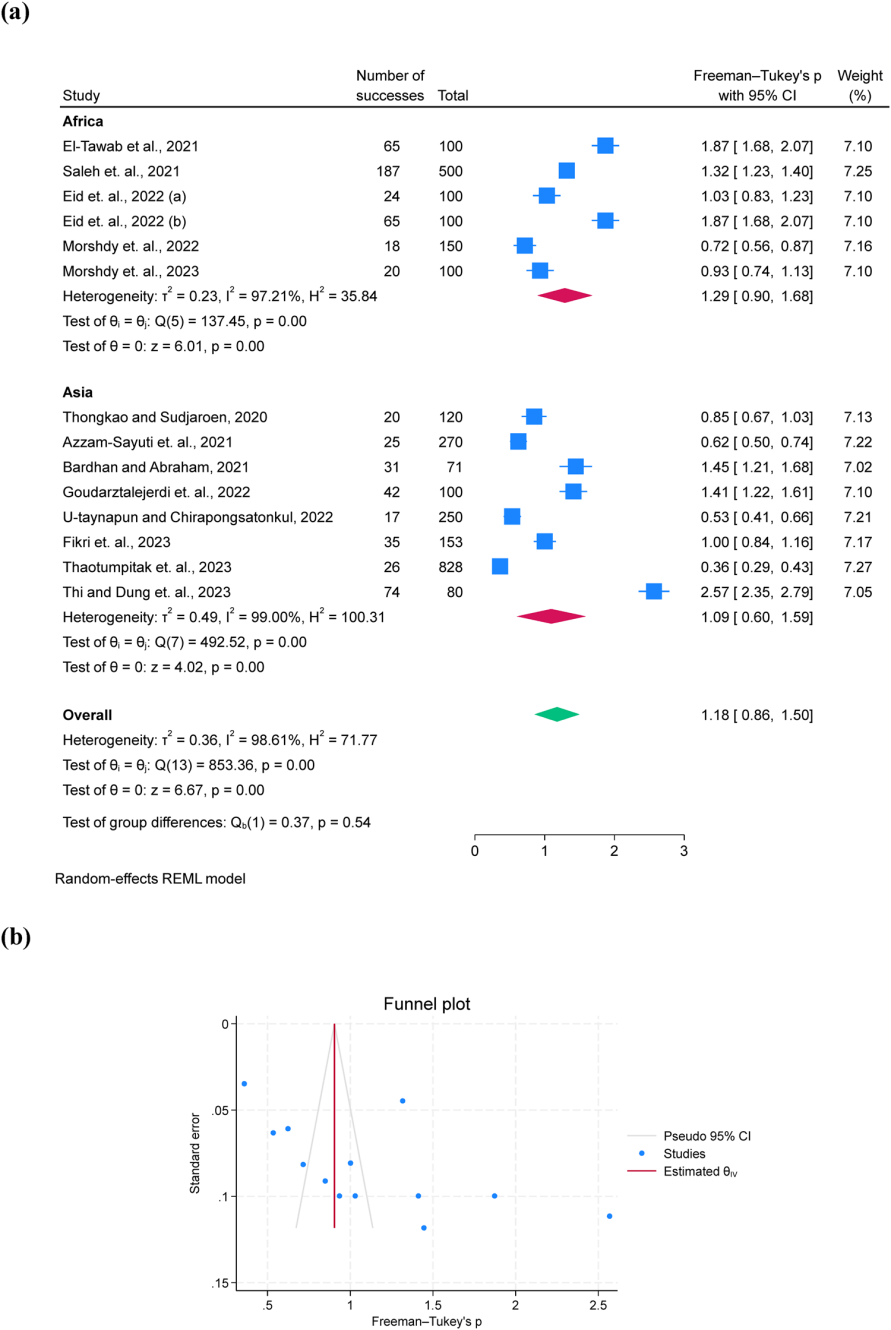
A forest plot comparing the prevalence differences of *A. hydrophila* between Africa and Asia **(a)** and a funnel plot for assessing publication bias **(b)**.

The prevalence of *A. hydrophila* was 30.7% with a 95% C.I. of 17.0-46.3% across 14 studies (Fig. 2). The subgroup by continent revealed a higher prevalence in Africa (36.0%; 95% C.I. = 18.6–55.5%, *I*^*2*^ = 97.21) compared to Asia (26.9%; 95% C.I. = 8.5–50.7%, *I*^*2*^ = 99.0) (Fig. 2a). A funnel plot for assessing publication bias presented fewer outliers (Fig. 2b).

### Pooled prevalence of *A. hydrophila* resistance

**Fig. 3:**
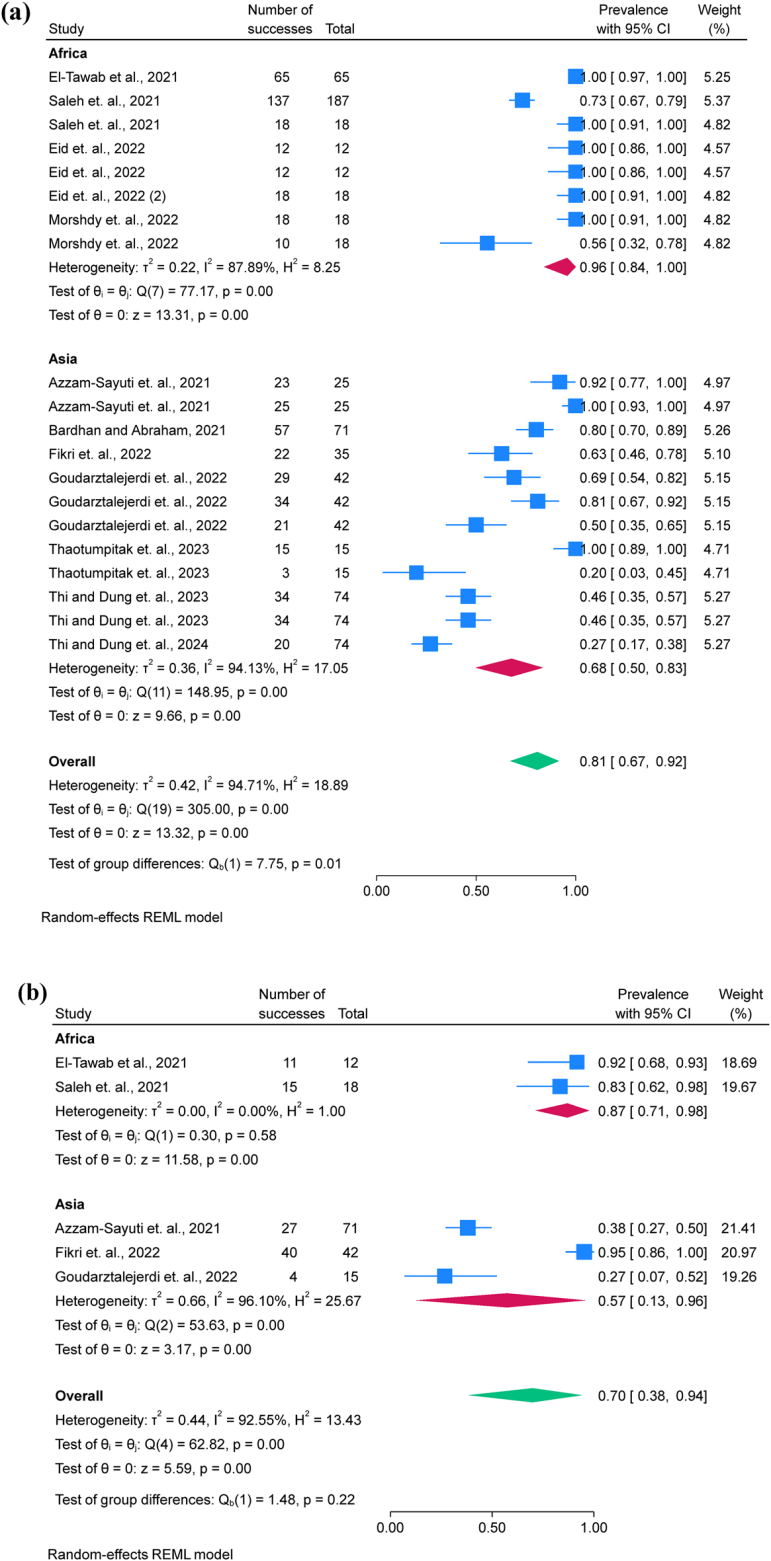

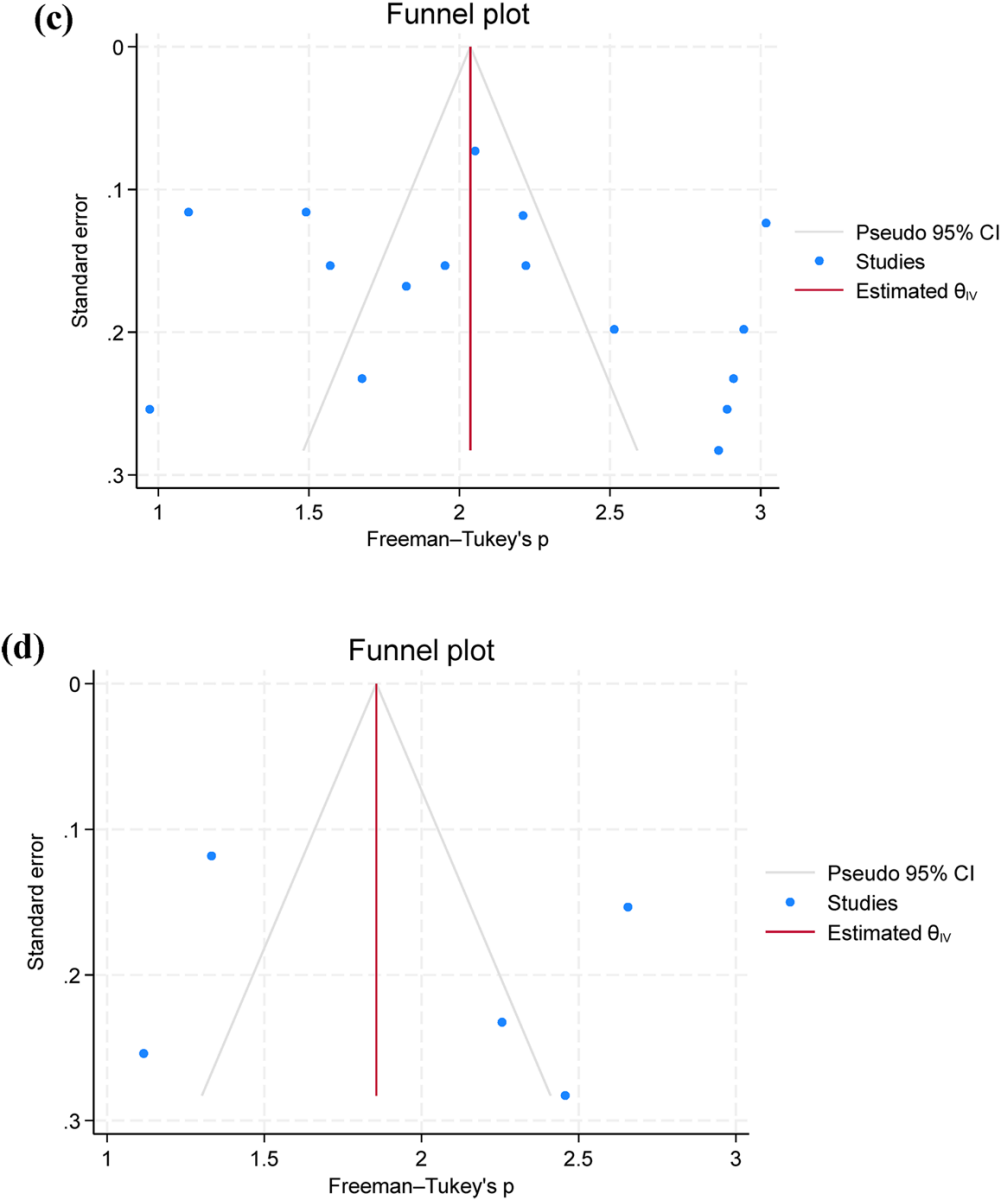
A forest plot comparing the differences in *A. hydrophila* resistance to penicillin **(a)** and oxytetracycline **(b)** between Africa and Asia, a funnel plot for assessing publication bias of the studies penicillin **(c)**, oxytetracycline **(d)**.

Twelve studies were included for the examination of AMR phenotypes, while two studies were excluded due to unclear reporting and low number of isolates (*n* = 4) used for AST^17,28^. AST was performed using 12 antimicrobial classes, including aminoglycosides, carbapenems, cephalosporins, chloramphenicol, penicillins, quinolones, lacosamide, nitrofuran, polymyxins, sulfonamides and tetracyclines. *A. hydrophila* showed the highest prevalence of resistance to penicillin (80.7%; 95% C.I. = 67.2–91.5%), oxytetracycline (69.9%; 95% C.I. = 38.5–94.0%), and macrolides (67.8%; 95% C.I. = 17.6–100%) and resistance was more frequently observed in Africa than in Asia (Fig. 3a, b, and c). Additionally, funnel plots were presented without outliers for the pooled prevalence of penicillin resistance, while one outlier was identified in oxytetracycline (Fig. 3d).

The highest prevalence of aminoglycoside resistance was found for streptomycin (39.0%; 95% C.I. = 13.2–68.3%), followed by amikacin (22.7%; 95% C.I. = 5.5–45.9%) and gentamicin (19.2%; 95% C.I. = 7.8–33.6%), while resistance to kanamycin and neomycin was reported in fewer studies (Tables 3 and 4). The pooled prevalence of various antimicrobial classes was as follows: penicillin (80.7%; 95% C.I. = 67.2–91.5%), macrolides (67.8%; 95% C.I. = 17.6–100%), carbapenems (44.0%; 95% C.I. = 4.7–88.5%), chloramphenicol (30.2%; 95% C.I. = 12.4–51.6%), cephalosporins (24.0%; 95% C.I. = 10.3–40.9%), and quinolones (16.8%; 95% C.I. = 5.9–21.2%). Additionally, one study observed the absence of Extended-Spectrum β-Lactamases (ESBL) production in *A. hydrophila*^29^. For sulfonamides, resistance was relatively high for sulfamethoxazole (57.5%; 95% C.I. = 22.7–88.7%) and trimethoprim (28.2%; 95% C.I. = 9.2–52.1%). In tetracyclines, resistance was relatively high for oxytetracycline (69.9%; 95% C.I. = 38.5–94.0%) and tetracycline (60.7%; 95% C.I. = 35.2–83.5%). Clindamycin, kanamycin, co-trimoxazole, doxycycline, nitrofurantoin, and colistin were each reported in only a single study. Additionally, the multiple antibiotic resistance (MAR) index, a ratio calculated by dividing the number of antibiotics an isolate is resistant to by the total number of antibiotics tested, The MAR index was found in 6 out of 12 studies (50%), with values ranging from 0.2 to 1.0.

**Table 3:** Summary of overall prevalence of AMR *A. hydrophila* stratified by antimicrobial class.

Antimicrobial class	Stratum	Isolate (*n*)	Prev (%)	95% C.I.	tau^2^	I^2^ (%)	H^2^ (%)	*p*-value
Aminoglycosides Amikacin	Africa Asia Overall	302 235 67	38.3 3.4 22.7	20.3–57.8 0-23.1 5.5–45.9	3.309	91.7	12.1	< 0.0001
Gentamicin	Africa Asia Overall	300 262 562	20.2 16.2 19.2	5.4–40.5 1.5–39.2 7.8–33.6	0.17	86.8	7.6	< 0.0001
Streptomycin	Africa Asia Overall	77 142 219	45.6 24.9 39.0	17.8–74.9 0-97.2 13.2–68.3	0.47	94.0	16.8	< 0.0001
Carbapenems*	Africa Asia Overall	205 77 282	77.2 14.3 44.0	36.3–100 0-73.1 4.7–88.5	0.94	97.9	47.3	< 0.0001
Cephalosporins*	Africa Asia Overall	523 366 889	31.8 17.8 24.0	13.8–52.7 1.6–43.6 10.3–40.9	0.50	96.1	25.6	< 0.0001
Chloramphenicol*	Africa Asia Overall	217 351 568	37.1 27.9 30.2	8.6–71.2 6.9–55.3 12.4–51.6	0.47	95.8	23.5	< 0.0001
Penicillin*	Africa Asia Overall	348 534 882	95.8 67.8 80.7	84.2–100 49.9–83.5 67.2–91.5	0.42	94.7	18.9	< 0.0001
Macrolides*	Africa Asia Overall	18 106 124	100 44.0 67.8	90.7–100 12.2–78.8 17.6–100	0.80	96.6	29.7	< 0.0001
Quinolones*	Africa Asia Overall	348 573 921	37.7 7.1 16.8	17.9–59.6 0.1–21.2 5.9–21.2	0.51	95.8	23.9	< 0.0001
Sulfonamides Sulfamethoxazole	Africa Asia Overall	235 202 437	88.7 22.9 57.5	65.5–100 0-65.5 22.7–88.7	0.98	97.9	47.1	< 0.0001
Trimethoprim	Africa Asia Overall	0 163 163	28.2 28.2	9.2–52.1 9.2–52.1	0.21	88.6	8.8	< 0.0001
Tetracyclines Tetracycline	Africa Asia Overall	270 208 478	77.5 51.7 60.7	26.7–100 23.7–79.1 35.2–83.5	0.55	96.3	27.1	< 0.0001
Oxytetracycline	Africa Asia Overall	30 128 158	86.9 57.2 69.9	71.5–97.6 12.8–95.7 38.5–94.0	0.44	92.6	13.4	< 0.0001
Virulence genes* *act*; *aeroH*; *aer*; *aerA*; *ahyB*	Africa Asia Overall	992 597 1,589	41.6 83.1 71.2	15.5–70.0 72.6–91.8 57.4–83.4	0.61	96.2	26.1	< 0.0001
Sulfonamide resistance genes *sul2*	Africa Asia Overall	7 15 22	77.0 13.3 49.2	3.0-100 0.3–36.1 0-99.9	0.79	82.4	5.7	< 0.0001
Tetracycline resistance genes *tetA*	Africa Asia Overall	12 67 79	100 24.7 63.7	83.8–100 0-67.9 19.0-94.8	0.89	90.7	10.8	< 0.0001
ESBL genes *bla*_TEM_	Africa Asia Overall	12 57 67	100 15.0 67.0	83.3–100 0–69.0 13.6–100	1.108	92.1	12.6	< 0.0001

Prev: prevalence; *: Data for different antimicrobials were combined to assess the overall prevalence of resistance.

**Table 4:** List of antimicrobials and antimicrobial classes collected from this study.

Antimicrobial class	Antimicrobial
Aminoglycosides	amikacin; gentamicin; streptomycin
Carbapenems	imipenem; meropenem
Cephalosporins	cefotaxime; cefpodoxime; ceftazidime; ceftriaxone; cefalexin; cefepime; cephalothin
Chloramphenicol	chloramphenicol; florfenicol
Penicillin	amoxicillin; ampicillin; amoxicillin/clavulanic; oxacillin; oxolinic acid; penicillin
Macrolides	azithromycin; erythromycin
Quinolones	ciprofloxacin; enrofloxacin; levofloxacin; nalidixic acid; norfloxacin
Sulfonamides	sulfamethoxazole; trimethoprim
Tetracyclines	Tetracycline; oxytetracycline

MDR were mentioned in two studies from Thailand (*n* = 4/15) and Egypt (*n* = 11/14).

### Prevalence of genotypic resistance and virulence genes of *A. hydrophila*

**Fig. 4:**
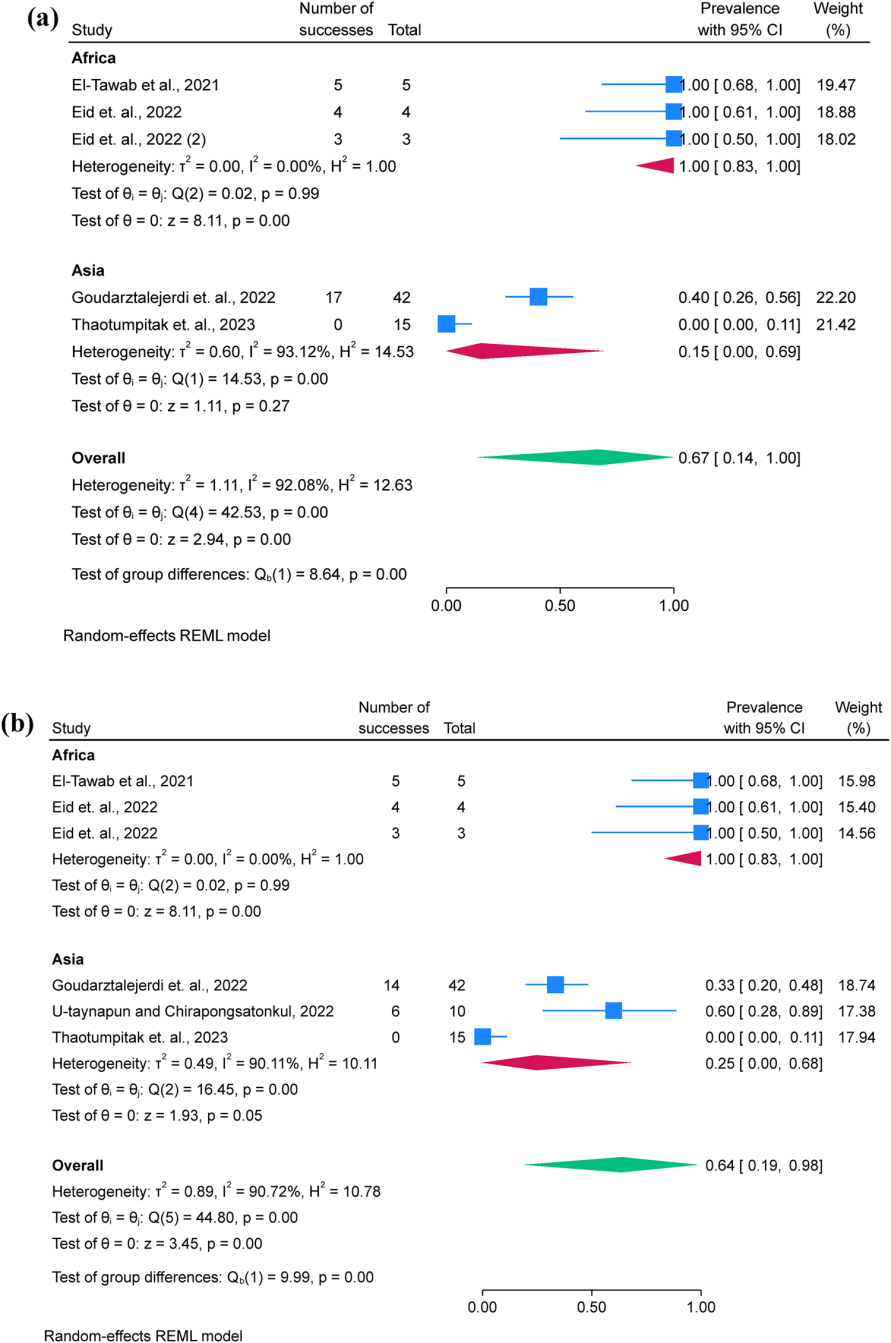

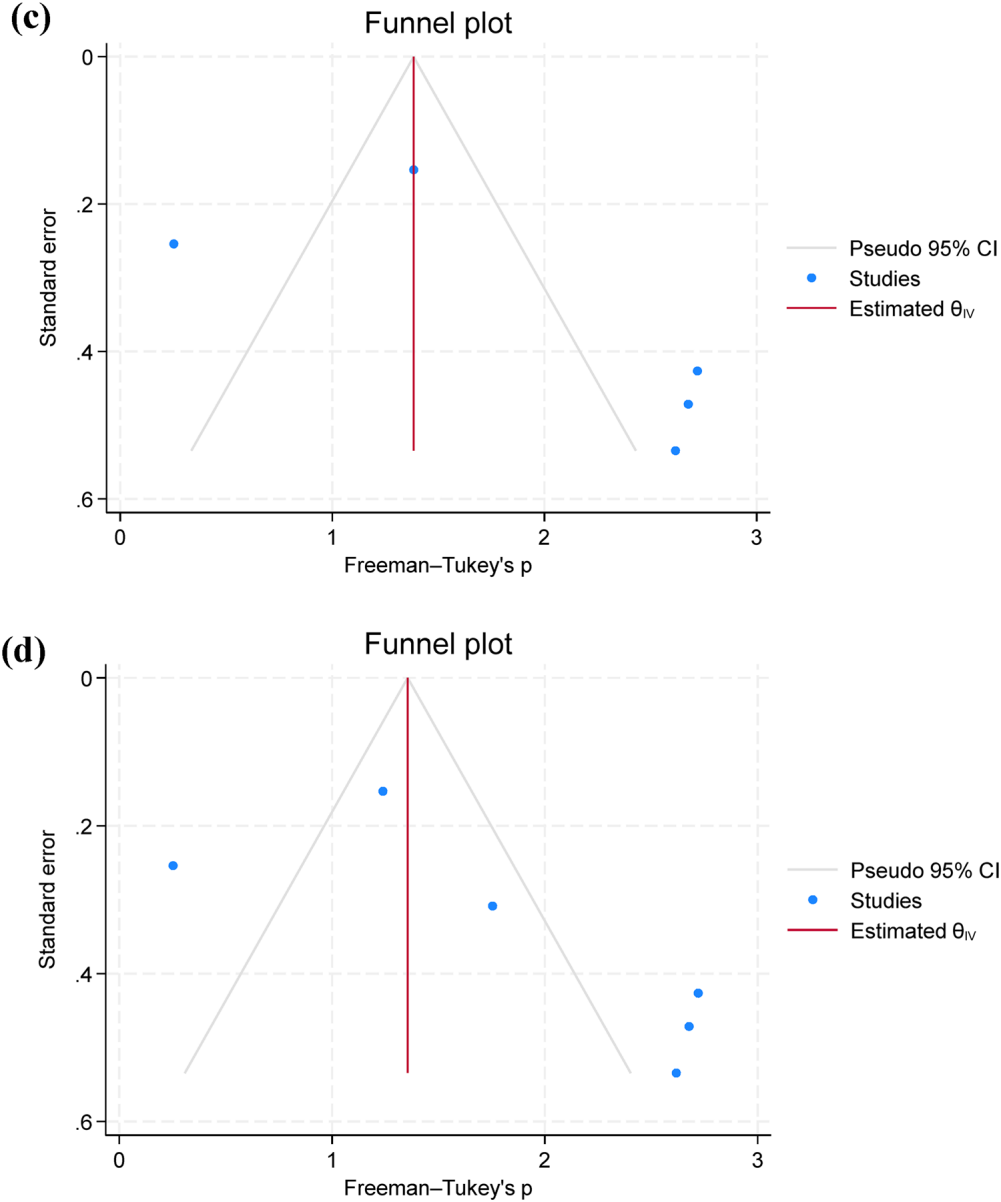
A forest plot comparing the differences in *A. hydrophila* resistance to *bla*_TEM_ **(a)** and *tetA* **(b)** between Africa and Asia, a funnel plot for assessing publication bias of the studies *bla*_TEM_ **(c)** and *tetA* **(d)**.

Only five studies assessed the pooled prevalence of virulence genes (*act*; *aer*; *aerA* and *ahyB*), with a pooled prevalence of 71.2% (95% C.I. = 57.4–83.4%). Additionally, resistance genes were limitedly assessed, with *bla*_TEM_ showing a prevalence of 67.0% (95% C.I. = 13.6–100%), followed by *tetA* 63.7% (95% C.I. = 19.0-94.8%) (Table 3; Fig. 4a and b). Additionally, funnel plots were presented without outliers for the pooled prevalence of *bla*_TEM_ and *tetA* (Fig. 4c and d).

Some AMR genotypes were infrequently observed in limited studies. Specific AMR genotypes observed include aminoglycosides (addA1; 5/5), trimethoprims (*dfrA1*: 3/3), colistin (*mcr-3*; 3/15), florfenicol (*floR*: 2/15), quinolones (*qnrS*: 2/15), sulfonamides (*sul2*: 2/15 and 10/42), and tetracyclines (*tetB*: 2/10; *tetC*: 5/10)^27^. Additional genes detected in a study by Thoathumpitak included *addA2*, *aac(3)IV*, *aac(6’)-Ib*, *bla*_SHV_, *bla*_CTX−M_, *bla*_NDM_, *bla*_PSE_, *bla*_OXA_, *catA*, *catB*, *cmlA*, *ermB*, *qepA*, *strA*, *strB*, *sul3*, *tetB*, *qnrB*, *dfrA12*, *mcr2-5*, *int3*, and *int*_SXT_, which were absent^29^. Integron (*int1*) was observed in only three studies: 0/15, 4/4, and 3/3, while integron (*int2*) was present in one study with a positive result of 2/42.

### Publication bias

As indicated by the funnel plot of the estimated prevalence of *A. hydrophila*, asymmetry was observed, suggesting potential publication bias (Smaller studies with positive outcomes seem to be published more frequently, as reflected in the upper left of the plot. To confirm this, an Egger’s test was performed to assess the statistical significance of the funnel plot’s asymmetry (Table 5). The Egger’s test revealed a significant small-study effect (*p* = 0.003), confirming the presence of publication bias. Similarly, publication bias was also observed in the prevalence of AMR to penicillin, *bla*_TEM_, and *tetA* (Table 5). However, no publication bias was detected in the pooled prevalence of oxytetracycline.

**Table 5:** Egger’s test for evaluating publication bias in the prevalence and phenotypic and genotypic AMR of *A. hydrophila*.

Observation	Beta 1	SE of beta 1	*P*-value
Prevalence of *A. hydrophila*	15.51	5.178	0.003
Resistance to penicillin	4.65	2.267	0.040
Resistance to oxytetracycline	1.27	5.717	0.825
Resistance to macrolides	10.42	12.188	0.392
Presence of *bla*_TEM_	5.33	2.683	0.047
Presence of *tetA*	5.56	2.683	0.014

### Quality assessment

The quality assessment revealed that 14 publications included in this study exhibited a low to moderate risk of bias. This suggests that the studies were generally of acceptable quality, with minimal concerns about systematic errors or biases that could affect the reliability of the results.

## Discussion

This systematic review and meta-analysis included a total of 14 studies, encompassing more than two thousand samples. The pooled prevalence of *A. hydrophila* was estimated to be almost one third (30.7%) among aquatic species, posing a significant threat to fish health and production. The study revealed the levels of AMR in *A. hydrophila*, particularly against oxytetracyclines. Moreover, *A. hydrophila* was widely distributed in aquatic environments and possessed several virulence factors that contribute to its pathogenicity^20^. In this study, the distribution of virulence was almost three-quarters, with the presence of five key virulence genes, including *act* (cytotoxic enterotoxin), *aeroH* (aerolysin), *aer* (aerolysin), *aerA* (aerolysin), and *ahyB* (elastase).

There is growing concern over the prevalence of *A. hydrophila* in aquaculture, which points to a significant disease threat. Specifically, the pooled prevalence of *A. hydrophila* in Africa was higher (36.0%) compared to Asia (26.9%). These findings were consistent with previous studies suggesting that environmental factors, such as water quality, aquaculture practices, and regional differences in pathogen exposure, may contribute to varying prevalence^31–33^. The higher prevalence observed in African studies could be attributed to specific environmental or ecological factors, including water contamination levels, aquaculture practices, and local pathogens. In contrast, Asian studies covered a broader range of countries, including those with advanced aquaculture systems, which might explain the slightly lower prevalence in this region^34,35^.

The most used antimicrobials in global aquaculture included tetracycline, oxytetracycline (tetracyclines), oxolinic acid, flumequine, sarafloxacin, enrofloxacin (quinolones), amoxicillin (β-lactam), erythromycin (macrolide), sulfadimethoxine (sulfonamide), ormetoprim (diaminopyrimidine) and florfenicol (amphenicol)^36^. A survey highlighted that oxytetracycline, florfenicol and trimethoprim/sulfadiazine were the most used antimicrobials on farms for disease control^37^. However, the improper use of these antimicrobials in managing diseases of farmed aquatic species posed a considerable threat to the sustainability of aquaculture industry. A major consequence of this was the risk of AMR, which complicates the treatment of common bacterial diseases in aquatic population.

The AMR profiles of *A. hydrophila* in this study revealed concerning levels of resistance to several antimicrobial agents, particularly penicillin (80.7%), oxytetracycline (69.9%), and macrolides (67.8%). This resistance was alarming, as they suggested that *A. hydrophila* might be capable of surviving and proliferating despite the use of common antimicrobials in aquaculture. Resistance to those antimicrobials was concerning because they were frequently used for the prevention and treatment of bacterial infections in aquatic animals. In general, high resistance of *A. hydrophila* is primarily due to intrinsic or chromosomally mediated resistance to ampicillin. However, significant resistance to oxytetracycline and macrolides is concerning, as these antimicrobials are commonly used in aquatic animals. The extensive use of antimicrobials has also led to their frequent detection in aquatic environments, negatively impacting water quality and ecological health. Water bodies near wastewater treatment plants (WWTPs) and densely populated areas were particularly contaminated, with macrolides being a significant pollutant^38^. Aquaculture systems and farms were marked as hotspots for AMR genes where significant genetic exchange and recombination could occur, shaping future resistance profiles^39^.

Surprisingly, resistance was found to be more frequent in Africa than in Asia. The widespread AMR observed in Africa, solely from Egypt, may be due to several factors, including the improper use of antimicrobials, limited stringent regulatory, and inadequate farm management practices. Additionally, differences in aquaculture practices across various Asian countries may influence the observed variations in *A. hydrophila* resistance. The presence of MDR strains in *A. hydrophila* was a critical finding, particularly given the rising prevalence of AMR in global aquaculture. These MDR strains posed a significant challenge to treatment options and could have had serious implications for public health and food safety. In Egypt and parts of Asia, the overuse and misuse of antimicrobials created selective pressure that promoted the development and spread of AMR bacteria. Additionally, environmental contamination through wastewater runoff and the proximity of aquaculture farms to urban areas could further facilitate the spread of AMR. Furthermore, the rise of extensive drug-resistant (XDR) and MDR strains was a significant concern, although only a few studies (*n* = 3) have identified MDR strains^22,25,29^. Moreover, a high MAR index (> 0.2), indicates a high-risk source of contamination. This index is a valuable tool for tracking the spread of MDR in epidemiology and public health surveillance. Resistant bacterial strains presented a serious threat to aquaculture and public health. The high prevalence of AMR phenotypes and genotypes in certain regions was primarily due to inadequate monitoring and control of antimicrobial use. To address this, it is essential to enforce stricter regulations on off-label antimicrobial use, implement better monitoring systems, and invest in research on alternative treatment options. These measures are vital for reducing the spread of AMR and safeguarding both animal and public health.

The prevalence of virulence genes in *A. hydrophila*, including was high, suggesting that these strains possessed strong pathogenic potential. These genes were associated with virulence factors such as motility, adhesion, and the ability to cause tissue damage, all of which were crucial for the bacterium’s ability to infect and survive in hosts. Additionally, the common resistance genes identified were with *bla*_TEM_ (67.0%), *tetA* (63.7%), and *sul2* (49.2%), which correspond to resistance mechanisms against ESBLs, tetracyclines, and sulfonamides, further supporting the notion that *A. hydrophila* was a significant threat in terms of both pathogenicity and resistance to multiple antibiotics. The presence of *bla*_TEM_ ESBL variants in aquaculture environments suggests the potential spread of ESBL-producing bacteria. In this study, the dissemination of *bla*_TEM_ genes in aquaculture was more prevalent in Africa compared to Asia. Conversely, *bla*_TEM_, and *bla*_SHV_ were the most frequently detected, with the highest number of AMR reports coming from Southeast Asia, particularly from Vietnam, Malaysia, and Thailand^40^. In contrast, ESBL-producing *Escherichia coli* genes were reported in various regions in South (68.0%), West (18.9%), Southeast (12.5%), and East (0.6%) with *bla*_CTX_-_M_ was more common gene detected^41^. However, the observed discordance between high phenotypic penicillin resistance and limited *bla*_TEM_ gene data in *A. hydrophila* is a common phenomenon in AMR studies and can be attributed to several factors, including the presence of other beta-lactamase genes (e.g., *bla*_CEP−H_ or *bla*_A1_), metallo-beta-lactamases (e.g., *bla*_CphA_), or other ESBL (e.g., *bla*_CTX−M_, *bla*_SHV_) that confer resistance against beta-lactams^42–45^.

Since ESBL genes are often carried on mobile genetic elements, such as plasmids, their transfer to other bacteria, including pathogenic species, is facilitated through horizontal gene transfer. This process contributes to the widespread dissemination of resistance determinants, even into pristine environments. The pathway of AMR in aquaculture involves the release of AMR genes into water systems and sediments, where they could spread through horizontal gene transfer and become integrated into the resistome^46^. The use of antimicrobials could drive the increasing prevalence of AMR genes in aquaculture and the environment. For example, *tetA* was identified to increase in the distal gut of fish and biofilms in tanks following oxytetracycline treatment^47^. Additionally, these genes (*bla*_TEM_, and *tetA*) were reported in animals, aquatic species, and humans highlighting the interconnected nature of AMR across species under One Health perspective^48^.

A key limitation of this study is that most of the data from Africa were solely from Egypt. This was because Egypt has seen significant growth in its aquaculture industry, making it the leading producer of aquaculture products in Africa. The country produced 1.6 million tons of fish annually, valued at USD 3.5 billion, with aquaculture accounting for 80% of fish production, primarily from private farms^48^. An uneven geographical distribution of data was noted within Asia and the scarcity of data from Europe and the United States. The scarcity of studies reporting on individual genes, such as *sul2* or virulence genes impacts statistical power, making their pooled prevalence estimates less reliable and generalizable. Additionally, the variability in AST methods, and the potential for misclassification bias in bacterial identification, particularly where studies relied solely on biochemical tests without molecular confirmation. Additionally, the pooled estimates for these specific outcomes should be interpreted with caution, recognizing the potential for inflated values.

We anticipate that future studies involving broader populations from Europe and the United States may show different prevalence and AMR genotypes and phenotypes. Another limitation of our study is the uncertainty regarding the completeness of resistance genotype prevalence reporting, as only a few studies were conducted on this aspect. To address this, we performed a quality assessment, and a funnel plot was presented with fewer outliers, suggesting low variability or heterogeneity among the studies. This indicates that the studies included in the analysis are relatively consistent, with no significant outlying results that could skew the overall findings.

## Conclusions

This systematic review and meta-analysis emphasized the growing concern of *A. hydrophila* and its AMR in aquatic food animals, posing significant risks to both animal and public health. The study highlighted the widespread prevalence of *A. hydrophila* and its resistance to commonly used antimicrobials, such as oxytetracycline, and macrolides. These findings offer important insights for healthcare professionals and policymakers by revealing the geographical distribution of resistant *A. hydrophila*, which could inform public health initiatives and priorities. The lack of effective surveillance and monitoring exacerbates the AMR issue, making it difficult to implement proper control and prevention efforts. To address this, stronger surveillance systems, stricter antimicrobial policies, and investment in alternative treatment research for aquaculture are essential. These measures are key to reducing the spread of AMR and protect both animal and public health.

## Supplementary Information

Below is the link to the electronic supplementary material.

Supplementary Material 1

Supplementary Material 2

## Data Availability

Data are provided within the manuscript or supplementary information files.
