# Supplementary material for: Global spread and antimicrobial resistance of Aeromonas hydrophila in aquatic food animals: a systematic review and meta-analysis
Source: Sci Rep. 2025 Aug 4;15:28441. doi: 10.1038/s41598-025-14498-8 (PMC12322169; doi:10.1038/s41598-025-14498-8)
Supplement: Supplementary file 1 — Supplementary Material 1 [file 41598_2025_14498_MOESM1_ESM.docx]

**Supplementary Table 2:** Risk of bias assessment of included studies using the Joanna Briggs Institute (JBI) critical appraisal checklist for studies reporting prevalence data

| **ID** | **Author** | **Year** | **Q1** | **Q2** | **Q3** | **Q4** | **Q5** | **Q6** | **Q7** | **Q8** | **Score** | **Overall**  **assessment** |
| --- | --- | --- | --- | --- | --- | --- | --- | --- | --- | --- | --- | --- |
| 1 | Abd El-Tawab et al. | 2021 | No | Yes | No | Yes | Yes | No | Yes | Yes | 7 | Low risk |
| 2 | Saleh et. al. | 2021 | No | Yes | No | Yes | Yes | No | Yes | Yes | 7 | Low risk |
| 3 | Eid et. al. (a) | 2022 | No | Yes | No | Yes | Yes | Yes | Yes | Yes | 8 | Low risk |
| 4 | Eid et. al. (b) | 2022 | No | Yes | No | Yes | Yes | No | Yes | Yes | 7 | Low risk |
| 5 | Morshdy et. al. | 2022 | No | Yes | No | Yes | No | No | Yes | Yes | 6 | Moderate risk |
| 6 | Morshdy et. al. | 2023 | No | Yes | No | Yes | Yes | No | Yes | Yes | 7 | Low risk |
| 7 | Thongkao and Sudjaroen | 2019 | No | Yes | No | Yes | Yes | No | Yes | Yes | 7 | Low risk |
| 8 | Azzam-Sayuti et. al. | 2021 | No | Yes | No | Yes | Yes | No | Yes | Yes | 7 | Low risk |
| 9 | Bardhan and Abraham | 2021 | No | Yes | Yes | Yes | Yes | No | Yes | Yes | 8 | Low risk |
| 10 | Goudarztalejerdi et. al. | 2022 | No | Yes | No | Yes | Yes | Yes | Yes | Yes | 8 | Low risk |
| 11 | U-taynapun and Chirapongsatonkul | 2022 | No | Yes | No | Yes | Yes | Yes | Yes | Yes | 8 | Low risk |
| 12 | Fikri et. al. | 2022 | No | Yes | No | Yes | Yes | Yes | Yes | Yes | 8 | Low risk |
| 13 | Thaotumpitak et. al. | 2023 | No | Yes | Yes | Yes | Yes | No | Yes | Yes | 8 | Low risk |
| 14 | Thi and Dung et. al. | 2023 | No | Yes | No | Yes | Yes | Yes | Yes | Yes | 8 | Low risk |

Note: Q1: Was the sample representative of the target population; Q2: Were study participants recruited in an appropriate way; Q3: Was the sample size adequate; Q4: Were the study subjects and the setting described in detail; Q5: Was the data analysis conducted with sufficient coverage of the identified sample; Q6: Were the objective, standard criteria used for measurement of the condition; Q7: Was the condition measured in a reliable way; Q8: Was appropriate statistical analysis used?
