# Supplementary material for: Global spread and antimicrobial resistance of Aeromonas hydrophila in aquatic food animals: a systematic review and meta-analysis
Source: Sci Rep. 2025 Aug 4;15:28441. doi: 10.1038/s41598-025-14498-8 (PMC12322169; doi:10.1038/s41598-025-14498-8)
Supplement: Supplementary file 2 — Supplementary Material 2 [file 41598_2025_14498_MOESM2_ESM.docx]

**Supplementary Table 1. Search strategy for systematic review on *A. hydrophila* in seafood**

| **Source** | **Search strategy** | **No. of**  **record** |
| --- | --- | --- |
| Scopus | TITLE-ABS-KEY ("*Aeromonas hydrophila*" OR "*A. hydrophila*") AND ("antibiotic" OR "antibiogram" OR "antimicrobial resistance" OR "resistance" OR "multidrug resistance" OR "antimicrobial susceptibility testing" OR "genotypic determinants" OR "genotype") AND ("virulence factors" OR "virulence genes") AND ("fish" OR "shellfish" OR "shrimp" OR "seafood") AND (PUBYEAR > 2019 AND PUBYEAR < 2025) | 909 |
| ScienceDirect | ("*Aeromonas hydrophila*" OR "*A. hydrophila*") AND ("antibiotic" OR "antimicrobial resistance" OR "resistance" OR "multidrug resistance" OR "antimicrobial susceptibility testing" OR "genotypic determinants" OR "genotype") AND ("virulence factors" OR "virulence genes") AND ("fish" OR "shellfish" OR "shrimp" OR "seafood") AND (publication year 2020-2024) | 2,854 |
| PubMed | (“*Aeromonas hydrophila*”[Title/Abstract] OR “*A. hydrophila*”[Title/Abstract]) AND (“antibiotic”[Title/Abstract] OR “antimicrobial resistance”[Title/Abstract] OR “resistance”[Title/Abstract] OR “multidrug resistance”[Title/Abstract] OR “antimicrobial susceptibility testing”[Title/Abstract] OR “genotypic determinants”[Title/Abstract] OR “genotype”[Title/Abstract]) AND (“virulence factors”[Title/Abstract] OR “virulence genes”[Title/Abstract]) AND (“fish”[Title/Abstract] OR “shellfish”[Title/Abstract] OR “shrimp”[Title/Abstract] OR “seafood”[Title/Abstract]) | 573 |
| Google Scholar | "*Aeromonas hydrophila*" OR "*A. hydrophila*" AND ("antibiotic" OR "antimicrobial resistance" OR "resistance" OR "multidrug resistance" OR "antimicrobial susceptibility testing" OR "genotypic determinants" OR "genotype") AND ("virulence factors" OR "virulence genes") AND ("fish" OR "shellfish" OR "shrimp" OR "seafood"). A custom date range of 2020–2024 was applied in Google Scholar’s advanced search options | 9,741 |
